# Cultural adaptation of psychological interventions for people with mental disorders delivered by lay health workers in Africa: scoping review and expert consultation

**DOI:** 10.1186/s13033-022-00526-x

**Published:** 2022-02-15

**Authors:** Dirceu Mabunda, Déborah Oliveira, Mohsin Sidat, Maria Tavares Cavalcanti, Vasco Cumbe, Flávio Mandlate, Milton Wainberg, Francine Cournos, Jair de Jesus Mari

**Affiliations:** 1 Department of Psychiatry, Federal University of São Paulo, São Paulo, Brazil; 2 Department of Community Health, Faculty of Medicine, Eduardo Mondlane University, Avenue Salvador Allende nr. 702, P.O Box: 1106, Maputo, Mozambique; 3 Instituto de Psiquiatria, Universidade Federal Do Rio de Janeiro, Rio de Janeiro, Brazil; 4 Department of Psychiatry, Columbia University College of Physician and Surgeons, New York, USA

**Keywords:** Lay health workers, Cultural adaptation, Africa, Psychological interventions

## Abstract

**Background:**

Lay Health Workers (LHW) are important providers of community mental health services and help mitigate access and treatment gaps in Africa. However, there is a paucity of knowledge about the role and performance of these workers, as well as about the extent to which the interventions delivered are culturally adapted to the African context.

**Aims:**

This scoping review aimed to explore the content and aspects concerning the cultural adaptation and sustainability of psychological interventions delivered by LHW to people with mental disorders in Africa.

**Methods:**

We conducted a scoping review of the peer-reviewed literature published from January 2000 to December 2018 to identify psychological interventions delivered by LHW for people with mental disorders in Africa. We systematically searched PubMed, Google scholar and Hinari to select relevant publications. The articles were evaluated for risk of bias according to study design with the National Heart, Lung, and Blood Institute’s (NHLBI) Quality Assessment Tools. Expert consultation was performed according to Arksey & O’Malley framework and cultural adaptation analysis was performed according to Bernal framework.

**Results:**

Out of 14,549 retrieved records, we identified ten peer-reviewed articles conducted in Zimbabwe, Uganda, South Africa and Zambia describing four distinct interventions. Six were randomized controlled trials; none addressed implementation outcomes. Group-based interpersonal therapy (n = 5), trauma-focused cognitive behaviour therapy (n = 1), problem solving therapy (n = 3) and narrative exposure therapy (n = 1) emerged as psychological interventions delivered by LHW for people with depression, anxiety, trauma and suicidal behavior. Psychological interventions delivered by LHW in Africa were all culturally adapted to meet the competence of LHW. All the interventions were associated with symptom improvement, but the quality of this evidence varied widely with study design.

**Conclusion:**

Task-shifting psychological interventions delivered by LHW after appropriate cultural adaptation show promise for addressing unmet mental health care needs in Africa. More effectiveness and implementation evidence is needed, especially with regard to psychological interventions delivered by LHW for adolescence, older people and those with severe mental disorders and suicidal behaviors.

## Introduction

In 2016, there were approximately one billion people living with mental illnesses globally [1, 2]. Almost 75% of these people live in low- and middle-income countries (LMIC) and this number is expected to increase [3]. Mental illnesses cost the global economy nearly US$2.5 trillion in 2010 [1, 4], with increased burden on governments and civil societies worldwide. Several psychological interventions have been shown to be effective and relevant to increasing service access and service use by people with mental illness in LMIC [5]. These interventions, however, need to be sensitive to the culture, values and needs of people locally [6, 7]. Community health workers, such as Lay Health Workers (LHW), are lay people with little or no formal education, but who have in-depth knowledge of the culture, beliefs and language of a community, and who receive short and standardized training to assist health professionals with the provision of culturally adapted and accepted health services [8]. LHW are individuals with little or no formal education who have undergone a few days to a few weeks of job-related pre-service training outside a recognised training institution [8, 9] using a task-shifting which is “*A process whereby specific tasks are moved, where appropriate, to health workers with shorter training and fewer qualifications… [to] make more efficient use of existing human resources and ease bottlenecks in service delivery*” [10]*.* Despite the importance of LHW to the delivery and sustainability of psychological interventions in LMIC, information about the role they perform, as well as the relevance and acceptability of the interventions they deliver, is currently scarce. Investigating the cultural relevance and sustainability of such interventions can help contribute towards the provision of effective mental health services in LMIC. These services, in turn, can contribute to achieving the United Nation’s Sustainable Developmental Goals and the World Health Organization’s Universal Health Coverage strategy in low-resourced settings, which include most African countries [11–13].

LHW provide numerous healthcare interventions in LMIC [14]. A multi-center qualitative study involving African and Asian nations (Ethiopia, Uganda, South Africa, India and Nepal) has shown that psychological interventions utilizing task-shifting techniques are acceptable in such contexts [15]. Other interventions delivered by LHW have also been shown to be effective. For example, a previous systematic review concluded that the feasibility of psychological interventions (cognitive behavioral therapy and interpersonal therapy) offered by LHW to women with perinatal depression was largely dependent on these therapies being delivered in community settings and on incorporating local social problems [16]. Another study conducted in China indicated that increasing resources for community health services through training and continued education can contribute towards reducing the gap in access to treatments for psychiatric disorders as well as reduce stigma [17]. In Uganda, a sustainable and inexpensive psychological model involving LHW helped reduce child mortality [18]. Even in high-income nations, such as the United States, research has shown that the work of LHW can be effective in helping communities recover from natural disasters [19]. In addition, community-based interventions delivered by LHW to people with schizophrenia in India, have shown greater effectiveness in reducing disability and increasing adherence to treatment compared to community-based treatment provided by health professionals [20].

Despite the important role that LHW play in the delivery of complex psychological interventions to people with mental illnesses in LMIC, the extent to which such interventions are implemented, culturally adapted and integrated within health systems remains under researched [5, 21]. In addition, previous research on the interventions delivered by LHW have not addressed issues that are important to policy makers, such as implementation and sustainability issues [22]. Sustainability as the ability to maintain the use of program at sufficient intensity to achieve its benefits over time [23, 24] contribute to long-term services provision, community support and ethical standards maintenance of the evidence-based interventions in different economic, cultural and social context [25].

Studies recommend that cultural adaptation of interventions is important to ensure acceptability, feasibility and effectiveness [7, 26]. This scoping review aimed to explore the content and aspects concerning the cultural adaptation of psychological interventions delivered by LHW to people with mental disorders in African countries. This is important to provide a broad overview on the existing knowledge concerning cultural adaptation of psychological interventions delivered by LHW in this context. We sought to answer three main questions: (a) What is the type and content of psychological interventions delivered by LHW in Africa countries; (b) Were such interventions culturally adapted and what elements were taken into account in the cultural adaptation process; and (c) Do the outcomes of these interventions look promising and sustainably?

## Methods

This scoping review was conducted in line with Arksey & O’Malley’s framework [27, 28] following six sequential steps: (1) identifying the research questions; (2) identifying relevant studies; (3) study selection; (4) charting the data; (5) collating, summarizing, and reporting the results; and (6) expert consultation. We also used the Bernal cultural adaptation framework to inform and to answer the second review question regarding cultural adaptation of interventions [29]. This framework has eight elements: (1) Language—culturally syntonic and appropriate language; (2) People—role of ethnical/racial differences and similarities between client and therapist in therapy relationship; (3) Metaphors—symbols and concepts shared with the population, sayings in treatment; (4) Content—cultural knowledge, values, traditions, uniqueness of groups (social, economic, historical, political); (5) Concepts—treatment concepts consonant with culture and context: dependence vs. interdependence vs. independence, emic, over ethic; (6) Goals—transmission of positive and adaptative cultural values, support adaptative values form the culture of origin; (7) Methods—development and/or cultural adaptation of treatment methods e.g., use of language (formal and informal); (8) Context—consideration of changing in assessment during intervention: acculturative stress, phase of migration, social support and relationship to country of origin, economic and social context of intervention; and (9) Security—humanitarian/conflict context in which client and intervention embedded; specific adaptations made as a result of strategies to manage risk.

To evaluate the capacity of the interventions for sustainability, we used the Program Sustainability Assessment tool framework (PSAT) [30] which has eight domains: (1) Environmental Support, (2) Funding Stability, (3) Partnerships, (4) Organizational Capacity, (5) Program Evaluation*,* (6) Program Adaptation*,* (7) Communications, and (8) Strategic Planning. There are 40 questions total (5 questions in each of 8 domains). For each question, participants are asked to rate the program on the presence of a particular element by using a scale of 1 (to little or no extent) to 7 (to a great extent) [31]. Two researchers (DM and FM) complete independently the PSAT assessment available online at https://sustaintool.org for each one of the 10 studies included. The site automatically calculate the average responses for each question.

### Search strategy

The literature search was conducted by two people independently (DM and FM), from January to February 2019. The search was conducted in the following electronic databases: Pubmed, Hinari, and Google scholar. The search strategy was guided by the review questions and included the following keywords and combinations: [("community health worker" OR "lay health worker" OR "community volunteer" OR "community activist" OR "traditional birth attendants" OR "health extension" OR "health extenders" OR "task shifting" OR "field worker" OR "close to community care" OR "outreach") AND ("psychology" OR "psychiatry" OR "mental health" OR "psychosocial" OR "counsellor" OR "counseling support") AND (Africa)]. The search strategy was adapted to the requirements of each database, using appropriate Boolean operators and coding, in order to increase search sensitivity. The references of the included papers were also checked for additional studies that might be relevant to this review.

### Study selection

#### Eligibility criteria

We limited our search to consider all the papers published in peer reviewed journals dated from January 2000 to December 2018. No inclusion/exclusion criterion for study design or specific methodology was used. Only original and peer-reviewed research papers published in English were included. Studies of any design reporting on the delivery of any psychological intervention by LHW in any African country were eligible. Eligibility was also dependent upon the paper including a description of the type of intervention and target population in sufficient detail and having elements of cultural adaptation described. Literature reviews, commentaries, editorials, program reviews, opinion papers, dissertations, and conference abstracts were excluded. Publications that focused on psychological interventions delivered by mental health professionals and articles that did not report any delivered intervention were excluded.

#### Procedure

**Fig. 1:**
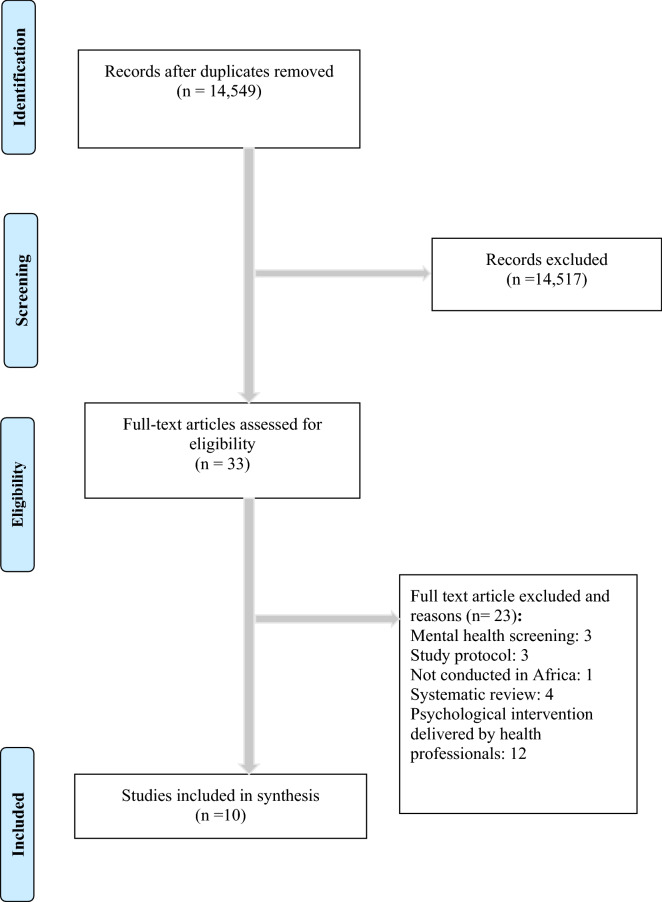
Scoping process flow diagram showing the number of studies identified and selected for inclusion. Developed using PRISM diagram

Study selection was conducted by two researchers independently (DM and FM) (Fig. 1). Titles and abstracts were examined against the review questions and eligibility criteria. A third reviewer (VC) was consulted in case of disagreement between the two reviewers regarding study eligibility. Over 93% of screened articles were excluded, mainly because they were not focused on psychological interventions delivered by LHW in Africa, and 6% of the papers were excluded because they reported on psychological interventions that were not delivered by LHW. After initial review, 32 articles were deemed potentially relevant and were read in full. On final review, ten of the 32 articles were deemed eligible and underwent data extraction.

#### Data extraction and synthesis

A table for data extraction was created and included: author(s) name(s), year of publication, study location, intervention type, study populations, study aim, methodology, outcome measures, key findings, and funding. After careful review of the included papers, two researchers (DM and FM) independently extracted data from the 10 (ten) studies. The findings from the expert consultation were collated and discussed between the research team, and were compared with the articles identified in the literature search. A narrative approach was used to discuss the review findings and the expert comments.

### Quality assessment

Each article that met the inclusion criteria was independently rated for quality by two researchers (VC and MT) using National Heart, Lung, and Blood Institute (NHLBI) quality assessment tools (https://www.nhlbi.nih.gov/health-topics/study-quality-assessment-tools) with separate tool for each study type. If the ratings differed, the third researcher (DM) reviewed the articles quality to reach consensus. The answer to each question can be “yes,” “no,” “cannot determine,” “not reported,” or “not applicable.” The rater is asked to consider potential risk for bias in the study design whenever a “no” is selected. Overall quality ratings are scored as “good” (low risk of bias, valid results), “fair” (some risk of bias, does not invalidate results), or “poor” (significant risk for bias, may invalidate results).

#### Expert consultation

We consulted ten clinical and research experts in the fields of LHW and global mental health about the topic of interest. Experts were identified from the literature in the area and were invited to complete an online questionnaire containing four open questions about gaps and strengths of cultural adaptation of psychological interventions delivered by LHW, how to address possible barriers, and the factors that could help sustain such interventions. Participants were research psychiatrists (n = 4) and psychologists (n = 3) from South Africa, Argentina, United States, and India. Four experts (n = 4) were representatives of the World Health Organization.

## Results

Key information about the ten included articles is shown in Table 1. The studies were published between 2003 and 2018, with the majority (70%) published after 2010.

**Table 1:** Summary of psychological interventions delivered by Lay Health Workers in Africa

Authors	Country	Setting	Objective of study	Intervention	Type of study	Sample size	Results/outcome
Petersen et al. 2011	South Africa	Rural	To assess the feasibility of the adapted IPT intervention for women with depressive symptoms that could be delivered by trained lay health workers within a task shifting approach	Grouped-based interpersonal therapy	Non-randomized, intervention	42 people: 20 intervention; 22 control	Decrease of mean beck depression inventory at 12- and 24-weeks post intervention
Bolton et al. 2007	Uganda	Rural	To assess effect of locally feasible interventions on depression, anxiety, and conduct problem symptoms among adolescent survivors of war and displace- ment in northern Uganda	Group interpersonal therapy	Randomized clinical trial	314 adolescents: 105 randomized to receive creative play; 105 randomized to receive Group IPT and 104 randomized to control group	Substantially and significantly greater decline in the depression symptom scale among the IPT group compared to wait-list control group. Difference in change in adjusted mean score for depression symptoms between group interpersonal psychotherapy and control groups was 9.79 points [95% confidence interval (CI), 1.66–17.93]. This substantial and significant improvement in depression symptoms (12.61 points; 95% CI 2.09–23.14) were found in girls but not in boys receiving group interpersonal psychotherapy
Verena et al. 2011	Uganda	Rural	To assess the efficacy of a community-based intervention targeting symptoms of posttraumatic stress disorder (PTSD) in formerly abducted individuals	Narrative exposure therapy	Randomized controlled trial	85 male and female former children soldier (from 12 to 25 years old) with positive screening result for PTSD	PTSD symptom severity (range: 0–148) was significantly more improved in the narrative exposure therapy group than in the academic catch-up [mean change difference, − 14.06 (95% confidence interval, − 27.19 to − 0.92)] and waiting-list [mean change difference, − 13.04 (95% confidence interval, − 26.79 to 0.72)] groups. Narrative exposure therapy produced a larger within-treatment effect size (Cohen d = 1.80) than academic catch-up (d = 0.83) and wait-listing (d = 0.81)
Chibanda et al. 2011	Zimbabwe	Suburban	to gather preliminary data on the effectiveness of this intervention and to see if the intervention would be feasible, and if so to gather ideas about how best to implement it on a larger scale	Brief individual talking therapy based on problem-solving therapy	Non-randomized, intervention	320 people	After receiving between 3 and 6 sessions the mean score of the SSQ (Shona Symptom Questionnaire) dropped by 4.8 points to 6.5 (sd = 2.4) [t = 13.6 (p = 0.0087)]
Murray et al. 2013	Zambia	Urban and peri-urban areas	to evaluate the feasibility of implementing TF-CBT and the changes in trauma-related symptoms with OVC	Trauma focused-cognitive behaviour therapy	Cohort study- prospective	58	The average PTSD score after treatment (27.6) was significantly lower than the average pre-treatment score (67.7, p < 0.0001). The effect was similar among males and females. Significant reductions in severity of shame symptoms (p < 0.0001)
Petersen et al. 2014	South Africa	peri-urban	to evaluate the potential effectiveness of an adapted group-based HIV counsellor delivered intervention for treating depression in people living with HIV/AIDS	Group-based counseling intervention for depressed co-morbid HIV patient (adapted from group-based IPT)	Randomized controlled pilot study	76	Significantly great improvement on depression scores on the PHQ9 in the intervention group compared to the control group at 3-month follow-up (mean difference score of 8.53 in the intervention group compared to mean difference score of 4.12 in the control group at follow-up) [F (1, 32) = 23.88, p < 0.0001]. at post-test, a significant decline in the mean scores on the HSCL-25 (Hopkins Symptoms Checklist) was found for both groups (1.97 and 2.13 for the two groups), [F(1, 32) = 17.48, p < 0.0001]
Chibanda et al. 2016	Zimbabwe	Urban	To evaluate the effectiveness of a culturally adapted psychological intervention for common mental disorders delivered by LHWs in primary care	Problem solving therapy	Randomized clinical trials	573 randomized patients. Woman- 495; 521 completed follow-up at 6 months	Intervention group participants had fewer symptoms than control group participants on Shona Symptoms Questionnaire (SSQ-14) (3.81; 95% CI 3.28 to 4.34; 95% CI 8.33 to 9.47; adjusted mean difference, − 4.86; 95% CI − 5.63 to − 4.10; p < 0.001; adjusted risk ratio, 0.21; 95% CI 0.15 to 0.29; p < 0.001).intervention group participants also had lower risk of symptoms of depression (13.7%; ARR, 0.28; 95% CI 0.22 to 0.34; p < 0.001)
Bolton et al. 2003	Uganda	Rural	To test the efficacy of group interpersonal psychotherapy in alleviating depression and dysfunction and to evaluate the feasibility of conducting controlled trials in Africa	Group IPT	Randomized controlled trial	248 persons. 108 males and 116 females. Refuse 9, others died	decline in depression scores was substantially greater among the intervention groups. Mean changes were 17.47 and 3.55 for the intervention and control groups, respectively (11.59 and 2.38). Within the intervention groups, the mean change among men was 14.43 (95% CI 12.32–16.55) compared with 20.46 (95% CI 18.09–22.84) among women
Bass et al. 2006	Uganda	Rural	To assess the long-term effectiveness of Interpersonal Psychotherapy this over a subsequent 6-month period	Group IPT	Randomized controlled trial	216	The overall mean functions impairment score was about 4 points lower in the intervention group compared with control group 2 weeks after the intervention and about 5 points 6 months after the intervention (p < 0.0001)
Munetsi et al. 2018	Zimbabwe	Urban	To determine whether problem solving therapy delivered by lay health workers can reduce common mental disorder symptoms among people with suicidal ideation,	Problem solving therapy for suicide ideation	Quasi-experimental study	573 persons	At 6-months severity of common mental disorder symptoms were significantly less among participants whom received LHW Friendship Bench care than among control group. The adjusted mean difference in SSQ-14 score participants with suicidal ideation was-5.38 (95% CI − 7.85, − 2.90; p < 0.001) and among those with common mental disorder symptoms but no suicidal ideation the adjusted mean difference was -4.86 (95% CI − 5.86, -4.04; p < 0.001)

IPT, interpersonal therapy; CHW, Community Health Workers; TF-CBT, trauma-focused cognitive behavior therapy; OVC, orphans and vulnerable childrens

The main interventions delivered by LHW were trauma-focused cognitive behavior therapy, interpersonal group therapy problem solving therapy and narrative exposure therapy. Three studies were undertaken in Zimbabwe, four in Uganda, two in South Africa and one in Zambia. Six were randomized controlled trials (RCTs), three were feasibility/pilot studies, and one was a quasi-experimental study. Sample sizes varied from 76 to 573 individuals in the RCTs and from 24 to 573 individuals in the feasibility/pilot studies. None of the studies addressed implementation outcomes.

### Intervention type and effectiveness

#### Randomized trials

Four RCTs involved the delivery of group-based interpersonal psychotherapy for people with depression in Uganda and with depression comorbid with HIV in South Africa.

A group-based interpersonal psychotherapy (IPT) intervention with people with depression (n = 248) of both genders conducted in Uganda showed greater improvement in total depression symptoms among the intervention group compared to control group (mean change in depression scores in men was 14.43 compared with 20.46 among women in the intervention group) [32]. The interventions were delivered by a local person of the same sex as the group who had received 2 weeks of intensive training in IPT Group. The basic structure of IPT was retained but was simplified to be used by LHW. The main additions included: grief was called death of a loved one; disputes were called disagreements; transitions became life changes; and interpersonal deficits became loneliness and shyness. Another study in Uganda (n = 216) with adults with depression showed long-term effectiveness of interpersonal therapy six months after the intervention [33]. A group based IPT intervention with adolescent of both genders (n = 314) survivors of war in northern Uganda [34] showed substantially and significantly greater decline in the depression symptom scale among the IPT group compared to wait-list control group. Difference in change in adjusted mean score for depression symptoms between group interpersonal psychotherapy and control groups was 9.79 points [95% confidence interval (CI) 1.66–17.93]. This substantial and significant improvement in depression symptoms (12.61 points; 95% CI 2.09–23.14) were found in girls but not in boys receiving group interpersonal psychotherapy. This intervention was delivered by a local Ugandan of the same gender with no previous mental health or counselling experience, who spoke English and Luganda (the local language) and participated in intensive training of two weeks comprising didactic and roleplay techniques.

One RCT in South Africa [35] tested the effectiveness of Group based IPT for people living with HIV with depression (n = 76) showed significantly great improvement on depression scores on the Patient Health Questionnaire-9 (PHQ-9) in the intervention group compared to the control group at 3-month follow-up (mean difference score of 8.53 in the intervention group compared to mean difference score of 4.12 in the control group at follow-up) (F (1, 32) = 23.88, p < 0.0001). The intervention was delivered by two of the lay HIV counsellors from the clinic who were trained in the intervention took place over four days. The IPT approach was maintained and adapted to focus on poverty, grief, interpersonal conflicts and stigma.

One RCT tested the effectiveness of problem-solving therapy for common mental disorders (n = 537) in Zimbabwe [36]. The intervention had a significant effect on improving the symptoms of common mental disorders compared to enhanced usual care group measured by Shona Symptoms Questionnaire [37]. Another RCT (n = 85) delivering narrative exposure therapy to former child soldiers from 12 to 25 years old with symptoms of trauma in Uganda showed significantly higher levels of reduction in PTSD symptoms in the intervention group, as well as larger within-treatment effect size for narrative exposure (Cohen d = 1.80), compared to the academic (*F*1,234.1 = 5.21, *p* = 0.02) (Cohen d = 0.83) and waiting list (*F*1,228.3 = 5.28, *p* = 0.02) [38]. The Friendship Bench intervention were delivered by grandmothers who were trained for eight days. This covered lectures on common mental disorders using local language (kufungisisa—thinking too much), skills to identify common mental disorders using the Shona Symptoms Questionnaire and how to manage common mental disorders using simple psychoeducation and problem-solving therapy on the Friendship Bench and in client’s homes. Home visits included prayer.

#### Feasibility/pilot studies

Two studies were conducted with people aged ≥ 18 in Zimbabwe involving problem-solving therapy, one aimed at reducing suicidal ideation (n = 573) [39] and the other aimed at improving the symptoms of depression and other common mental disorders (n = 320) [40]. The first study was conducted with people who scored above 9 on the 14-point Shona Screening Questionnaire and answered yes to a question about thoughts of suicide in the past week. This study showed a significant decrease in suicidal ideation [39]. The second study showed a significant effect of trauma focused therapy on improving the severity of common mental disorders among the intervention group compared to the control group [40]. The intervention was delivered by grandmothers who were trained during 8 days in the Friendship Bench Intervention.

One study (n = 42) in South Africa showed a significant decrease in depressive symptoms at 12 and 24 weeks after group-based interpersonal psychotherapy (IPT-G) for women with depression compared to a control group [41]. The intervention was delivered by LHW on their home visitation programme, who provided counseling for HIV and was preceded by a 2-day training course in basic counseling skills. The South African adaptation was derived from an adaptation of IPT-G in Uganda. The adaptation of IPT include the following domains: grief (especially associated with multiple losses due to HIV/AIDS), interpersonal conflicts (particularly involving abuse), life transitions (specifically finding out and living with an HIV status), and financial stress.

Another study (n = 58) in Zambia showed that the implementation of a trauma-focused cognitive behavior therapy was feasible to implement and using of LHW to treat trauma and stress related symptoms in orphans and vulnerable children in Zambia [42]. The intervention was delivered by volunteer outreach workers. Training was provided to local counselors who had little to no mental health training background. Training and supervision followed a version of the Apprenticeship Model with cultural modifications. There was an initial in-person training, followed by practice groups where local counselors and supervisors role-played TF-CBT components based on agendas developed by trainers. Supervisors and counselors met locally 2–4 h a week, and trainers spoke with the local supervisors via meeting/skype/phone for at least 2 h a week per supervisor.

### Cultural adaptation

All studies relied on the cultural competency of the therapists (n = 10; 100%). Six studies (60%) incorporated language translation and local practices into treatment, seven (70%) incorporated religious concepts, seven (70%) incorporated community engagement and adaptation of methods and training to match LHW experiences, five (50%) incorporated use of local symbols, and six studies (60%) incorporated client derived goals. None of the studies incorporated safety training and safety protocols. This domain is associated with conflict affected contexts but also with all security issues resulted from humanitarian, climate change and other types of security risk. This domain is universal (e.g., Security issues of the COVID-19 should be addressed in all settings). See Table 2 for the details regarding how each study incorporated cultural adaptation. The interventions were first developed in United States of America (Interpersonal therapy, Trauma focused therapy), United Kingdom (Problem solving therapy) and Australia (Narrative exposure therapy).

**Table 2:** Cultural adaptations of interventions

Adaptation principle	Bolton et al. 2003. Uganda	Bass et al. 2006. Uganda	Bolton et al. 2007	Chibanda et al. 2011. Zimbabwe	Petersen et al. 2011. South Africa	Ertl et al. 2011. Uganda	Murray et al. 2013. Zambia	Petersen et al. 2014. South Africa	Chibanda et al. 2016. Zimbabwe	Munetsi et al. 2018. Zimbabwe
Where the Intervention were first developed	USA	USA	USA	UK	USA	Australia	USA	USA	UK	UK
Language (translation in local language)	+	+	+	+	−	−	−	−	+	+
People (cultural competences of therapists)	+	+	+	+	+	+	+	+	+	+
Metaphors (use of stories and local examples, use local symbols/idioms)	+	+	+	+	−	−	−	−	+	−
Content (incorporation of local practices into treatment)	+	+	+	+	+	−	−	+	+	+
Concepts (address stressors, social concepts, religious concepts)	+	+	+	+	+	−	−	+	+	+
Goals (clients-derived goals)	+	+	+	+	−	+	−	−	+	−
Methods (adaptation of training and supervision methods, i.e., to match LHWs experience levels)	+	+	+	+	−	+	−	−	+	+
Context: Ensure acceptability (community engagement, ensuring fit with health/illness narratives)	+	+	+	+	−	+	−	−	+	−
Security: Specific adaptation relating to conflict-affected setting (i.e., safety training, safety protocols)	−	−	−	−	−	−	−	−	−	−

### Sustainability factors

Descriptive data on implementation factors and sustainment outcomes of the 10 studies included are summarized in Table 3. The majority of the studies (8 of 10) reported average PSAT scores in the mid-level range (4.0–5.6). Funding stability and strategic planning were the challenging domains for the sustainability of studies in this review.

**Table 3:** Program sustainability assessment tool (PSAT) descriptive

PSAT domain	Minimum	Maximum	Mean	SD
PSAT average	3.4	5.6	4.4	0.64
Environmental support	3.0	5.6	4.42	0.75
Funding stability	2.2	5.0	3.34	0.74
Partnerships	2.2	6.2	4.52	1.22
Organizational capacity	3.8	5.8	4.78	0.68
Program evaluation	3.8	5.8	4.9	0.59
Program adaptation	3.8	5.8	4.46	0.68
Communications	3.8	6.4	4.9	0.77
Strategic planning	2.4	5.2	3.9	0.71

### Quality assessment

Few studies (*n* = 3, 30%) were determined to be of “good” quality using the NHLBI Quality Assessment Tools. The remaining studies were “fair” (*n* = 3, 30%) or “poor” (*n* = 4, 40%). A common weakness specifically for controlled intervention studies was a lack of participants and provider blinded to treatment group assignment. One RCTs did not provide any description for how participants were allocated. The outcome assessors in all RCTs were blinded. For pre post-tests, the studies did not include sample size justification, power description and only 1 of 3 clearly defined the outcome measures and implemented consistently across all study participants. Without sample size justification and outcome measures there is a less confidence that an improvement or change between pre and post assessment is due to intervention rather than chance or effect of difference among groups. The cross-sectional study had small sample size and without power description. Confounders were rarely measured and included in the analyses.

### Expert views

**Fig. 2:**
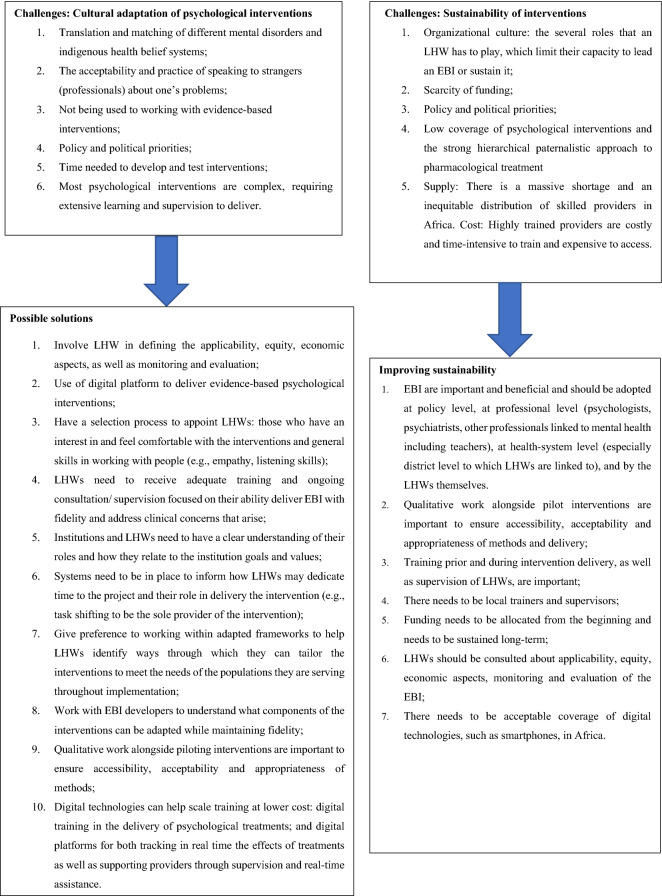
Expert suggestions

Overall, experts suggested that the cultural adaptation of psychological interventions delivered by LHW is limited because psychological interventions are complex, time-consuming, and require expertise and good supervision (Fig. 2). Experts recommended that research in this area should involve LHW in defining applicability, equity, monitoring evaluation and learning, from the adaptation, qualitative assessment, economic evaluation and scaling up. It is also important to work with the developers of evidence-based interventions to maintain their construct validity. The use of digital technology is necessary for real time training, supervision and remote treatment. Psychological interventions should be sustainable over time. Studies should include qualitative components to understand and ensure accessibility, acceptability and appropriateness of the contents. Ongoing training and supervision of LHW is needed and it’s important to develop local trainers and supervisors. Funding needs to be available from the beginning of the intervention delivery and should be sustained long-term.

## Discussion

Ten eligible studies from South Africa, Uganda, Zimbabwe and Zambia were analyzed with regard to the frequency with which cultural adaptations were utilized when introducing psychological interventions into LMIC in Africa with a plan to have them conducted by lay health workers. Five studies fulfilled the majority of the domains of the cultural adaptation framework of reference [29]. Our review suggest that psychological interventions delivered by LHW are effective and acceptable in sub-Saharan Africa, which is in line with other studies [13, 21, 43].

Results indicate mid-level sustainability (M = 4.4; SD = 0.64), which is similar to rate of study by Scuder [44]. In our review, financial support and strategic planning are two major challenging domains for the sustainability of the interventions delivered by LHW in Africa. Review by Iwelunmor [45] suggest that lack of funding is one of the barriers for sustainability of EBP interventions in Africa. Our study confirms the suggestion by Shelton [24] that strategic planning are critical to sustainment success.

Experts recommended that LHW should be involved in defining of applicability, monitoring and evaluation, participate in qualitative work and the use of digital technologies to facilitate the scale up of training and supervision at lower cost, this findings is in line with the study by Susan et al. [46]. Whilst the main objective of this review was to explore if the psychological interventions delivered by LHW in Africa were culturally adapted, the included papers were mostly focused on the intervention itself, with cultural adaptation only encompassing a small aspect. A cultural adaptation framework application was missing in most of the studies. Therefore, it was not possible to gain in-depth information on the cultural relevance and acceptability of each element such as format, content and delivery methods. In other LMIC, cultural adaptation includes shortening the time of training and delivery of the interventions, using local languages, integrating cultural and religious aspects and involving local LHW [47, 48]. These aspects were also observed in our results.

Experts recommended the use of digital technology to improve mental health care. Globally, digital technology tools applied to the screening, diagnosis, treatment, training and supervision, data management, online therapies, self-care, peer support and reduction of stigma, can improves LHWs effectiveness, the quality of care and adherence to protocol of the interventions [49, 50]. Mobile technology led to improvements in diabetes and other noncommunicable diseases control for a rural Guatemalan population [51]. African has the fastest growing mobile network and smartphone access, facilitating more people in the continent to use their mobile devices to access health care advice [52]. In rural Africa, studies have shown that use of mHealth technologies improves the perceived quality of care and increases adherence to health protocols [53]. Other study suggest that nonspecialized providers found the m-Screen Brief Intervention and Referral (m-SBIRT app) to be acceptable, appropriate, and feasible on task-shifting to expand mental health services [54].

Our review also suggests that the use of psychological interventions shows promise in addressing unmet mental health care needs in LMIC in Africa, but the reach and sustainability of these interventions need to be better addressed.

### Strengths and limitations

To our knowledge this review is the first to focus on the cultural adaptations of psychological interventions delivered by LHWs in Africa. Our literature searches were systematic and transparent but searching for available psychological interventions in Africa is complex because variety of linguistic and cultural background and we analyzed only the studies indexed in the chosen databases and published in English. Even though we tried to find papers across every country in the continent, only papers from four countries were included and therefore we can’t generalize our findings to the entire continent. It is a limitation of the study, not having other perspectives from social science experts.

Another limitation is the overall quality of evidence. “Poor” and “fair” quality ratings for the majority of studies make it difficult to make conclusions.

### Implications for future research

Future developers and implementers of LHW interventions should be aware of the importance of culturally adapted psychological interventions prior to implementation in different settings. Future research should focus on identifying barriers and facilitators to cultural adaptation according to LHW perspectives. We should seek to identify in-depth accounts of LHW experience and participation in cultural adaptation process, including aggregation of local priorities for the interventions, and this process should be more detailed in future publications in this area. A further review exploring the experiences of trainers and supervisors regarding cultural adaptation of these interventions is warranted. Synthesis of the experience and participation of LHW from conception to the delivery of psychological interventions can then inform the future development and transfer of cultural adaptation of psychological interventions. Future research should seek to answer critical questions regarding adoption and sustainability of the interventions in Africa.

## Conclusions

Psychological interventions delivered by LHW appear mostly to be culturally adapted to meet religious concepts and cultural competence of the LHW in African nations. Cultural adaptation of the interventions is an essential component for effective implementation and to guarantee the construct validity of the interventions for people with common mental disorders. This review highlights the important elements that implementers should be aware of when developing psychological interventions to be delivered by LHW. Moreover, we found a scarcity of evidence on psychological interventions delivered by LHW for adolescents, elderly and for people with severe mental disorders or suicidal behaviors.

## Data Availability

The data are available under reasonable request addressed to Dirceu Mabunda (dihepama@gmail.com).

## Abbreviations

LHW: Lay health workers
NHLBI: National Heart, Lung, and Blood Institute
LMIC: Low- and middle-income countries
RCTs: Randomized controlled trials
IPT-G: Group-based interpersonal psychotherapy
PSAT: Program sustainability assessment tool
PHQ-9: Patient health questionnaire-9
